# Sports-ACtrans Net: research on multimodal robotic sports action recognition driven via ST-GCN

**DOI:** 10.3389/fnbot.2024.1443432

**Published:** 2024-10-11

**Authors:** Qi Lu

**Affiliations:** Physical Education Institute, Henan Polytechnic University, Jiaozuo, Henan, China

**Keywords:** neural computing, computer vision, Swin Transformer, ST-GCN, reinforcement learning, multi-modal robot, sports action recognition

## Abstract

**Introduction:**

Accurately recognizing and understanding human motion actions presents a key challenge in the development of intelligent sports robots. Traditional methods often encounter significant drawbacks, such as high computational resource requirements and suboptimal real-time performance. To address these limitations, this study proposes a novel approach called Sports-ACtrans Net.

**Methods:**

In this approach, the Swin Transformer processes visual data to extract spatial features, while the Spatio-Temporal Graph Convolutional Network (ST-GCN) models human motion as graphs to handle skeleton data. By combining these outputs, a comprehensive representation of motion actions is created. Reinforcement learning is employed to optimize the action recognition process, framing it as a sequential decision-making problem. Deep Q-learning is utilized to learn the optimal policy, thereby enhancing the robot's ability to accurately recognize and engage in motion.

**Results and discussion:**

Experiments demonstrate significant improvements over state-of-the-art methods. This research advances the fields of neural computation, computer vision, and neuroscience, aiding in the development of intelligent robotic systems capable of understanding and participating in sports activities.

## 1 Introduction

Robot sports action recognition is a significant research direction in computer vision and machine learning, combining robotic capabilities with sports activities to achieve accurate understanding and recognition of human sports actions (Hong et al., 2024). This research holds considerable importance in promoting the application of intelligent robots in sports, including aiding training, teaching, and participating in sports competitions (Psaltis et al., 2022). This article introduces five commonly used deep learning or machine learning models in the field of robot sports action recognition and discusses their advantages and disadvantages (Ai et al., 2023).

Convolutional Neural Networks (CNN) (Wang, 2024) is a classic deep learning model widely used in image recognition tasks. It leverages local receptive fields, weight sharing, and pooling operations to capture spatial features in images. However, for robot sports action recognition, CNNs have limitations in handling temporal information and joint movements. Keshun et al. (2023b) Recurrent Neural Networks (RNN) (Baradel et al., 2017) is a deep learning model suitable for sequential data processing. It uses recurrent connections to handle temporal information and can capture the evolution of action sequences. However, traditional RNNs face issues like vanishing and exploding gradients, limiting their ability to model long sequences effectively. Long Short-Term Memory Networks (LSTM) (Imran and Raman, 2020) is an improved variant of RNN that introduces gating mechanisms to address the vanishing and exploding gradient problems. You et al. (2023) It excels in processing long sequences and modeling temporal relationships. However, LSTM still has drawbacks, such as a large number of parameters and computational complexity. Spatial Temporal Graph Convolutional Network (ST-GCN) (Duhme et al., 2021) is a graph convolution-based method specifically designed for skeleton data. It models skeleton data as a graph structure and uses graph convolution operations to capture spatial and temporal relationships between skeletal joints. ST-GCN has achieved remarkable results in action recognition tasks, but for robot sports action recognition, relying solely on skeleton data might not fully exploit the available visual information. Transformer (Jiang and Lu, 2023), initially proposed for natural language processing tasks, have also made significant strides in computer vision, such as with the Swin Transformer. Transformers use self-attention mechanisms to capture global dependencies in images, making them suitable for handling image sequences and extracting spatial features.

Current robot sports action recognition methods predominantly rely on single-modal data, such as images or skeleton data, which limits their potential. Multi-modal data, by contrast, offers a more comprehensive description of sports actions through richer information. Our motivation is to integrate multi-modal information by combining visual and skeleton data to enhance performance in robot sports action recognition. For the visual modality, we use the Swin Transformer to process video frame data. The Swin Transformer employs self-attention mechanisms to establish global dependencies in images and extract spatial features, serving as the visual feature representation. For the skeleton modality, we use the Spatial Temporal Graph Convolutional Network (ST-GCN) to process skeleton data. ST-GCN models the skeleton data as a graph structure and uses graph convolution operations to capture spatial and temporal relationships between skeletal joints, providing the skeleton feature representation. We design a multi-modal fusion architecture to integrate visual and skeleton features. Fusion can occur at the feature level or the decision level, creating a comprehensive representation of sports actions. This approach leverages the complementarity of multi-modal data, enhancing accuracy and robustness. To further optimize action recognition performance, we introduce reinforcement learning techniques. By modeling the task as a sequential decision problem and using algorithms like deep Q-learning or policy gradients, we optimize the system. Appropriate reward functions are employed to maximize expected rewards, improving the accuracy and stability of action recognition.

- We propose a method that integrates visual and skeleton data to achieve a comprehensive description of robot sports actions.
- We innovatively apply deep learning models such as the Swin Transformer and ST-GCN to effectively extract visual and skeleton features, capturing the spatio-temporal relationships of actions.
- By introducing reinforcement learning techniques, we optimize action recognition performance and stability. Modeling the recognition task as a sequential decision problem and maximizing expected rewards makes the recognition system more intelligent.

## 2 Related work

### 2.1 Representation learning

With the development of machine learning and deep learning, multi-modal data fusion and representation learning have become popular research directions in the field of robot sports action recognition (Wu et al., 2022). Multi-modal data fusion aims to effectively combine data from different sensors or modalities to improve action recognition performance. Representation learning focuses on finding suitable feature representations to fully utilize the complementarity of multi-modal data. Keshun and Huizhong (2023) In the aspect of multi-modal data fusion, researchers have explored different fusion strategies, such as feature-level fusion, decision-level fusion, and model-level fusion. Feature-level fusion obtains fused feature representations by concatenating or weighted summing features from different modalities. Decision-level fusion integrates classification results from different modalities, such as through voting or weighted averaging. Model-level fusion jointly trains or fuses network models of multiple modalities (Zhang et al., 2023). Research in representation learning mainly focuses on how to learn more discriminative and robust feature representations. Methods such as autoencoders, Generative Adversarial Networks (GANs), and Variational Autoencoders (VAEs) in deep learning have been widely used for representation learning of multi-modal data (Islam and Iqbal, 2021). These methods, through unsupervised or semi-supervised learning, learn feature representations with strong representational capabilities, thereby improving action recognition performance. Researchers have also proposed many innovative multi-modal datasets and evaluated different fusion and representation learning methods through experiments on these datasets (You et al., 2022).

### 2.2 Action recognition

Transfer learning and cross-domain action recognition are critical research directions in the field of robot sports action recognition, aiming to address action recognition problems across different scenarios and tasks (Wang et al., 2024b). Transfer learning improves action recognition performance in the target domain by leveraging knowledge and models from the source domain (Chen et al., 2024). Cross-domain action recognition involves transferring action recognition models from one domain to another to achieve knowledge transfer and application. Transfer learning methods mainly include feature transfer, model transfer, and relation transfer. Feature transfer applies feature representations from the source domain to the target domain, utilizing source domain knowledge to aid action recognition in the target domain. Model transfer involves applying model parameters from the source domain to the target domain's model training to reduce the need for samples and training time in the target domain. Relation transfer establishes relationships between the source and target domains to achieve knowledge transfer and migration (Keshun et al., 2023c). Future research can further explore the application of transfer learning and cross-domain action recognition methods in robot sports action recognition (Chao et al., 2024). This can include studying how to select the most appropriate source and target domains and how to design effective knowledge transfer strategies. Additionally, combining multi-modal data fusion and representation learning methods can further improve the performance of transfer learning and cross-domain action recognition (You et al., 2023).

### 2.3 Online learning and incremental action recognition

Online learning and incremental action recognition are cutting-edge research directions in the field of robot sports action recognition, aiming to achieve rapid learning and adaptation to new actions (Keshun et al., 2024a). Online learning refers to the real-time learning and updating of action recognition models through interaction with the environment during the actual operation of the robot. Incremental action recognition involves quickly learning new actions based on existing models, achieving incremental updates and expansions of the model (Liu et al., 2023). Online learning methods mainly include reinforcement learning, online supervised learning, and active learning. Reinforcement learning optimizes the action recognition model by obtaining reward signals through interaction with the environment. Online supervised learning continuously updates model parameters by collecting labeled data online. Active learning optimizes the learning process by actively selecting samples (Wang et al., 2024a). Incremental action recognition methods include incremental learning, transfer incremental learning, and deep incremental learning. Incremental learning involves adding new samples and labels based on existing models to achieve incremental updates and expansions of the model (Keshun et al., 2024b). Transfer incremental learning transfers knowledge from existing models to new action recognition tasks to achieve rapid learning and adaptation. Deep incremental learning combines deep learning models with incremental learning methods to achieve quick learning and recognition of new actions (Keshun et al., 2023a).

## 3 Methodology

### 3.1 Overview of our network

**Figure 1:**
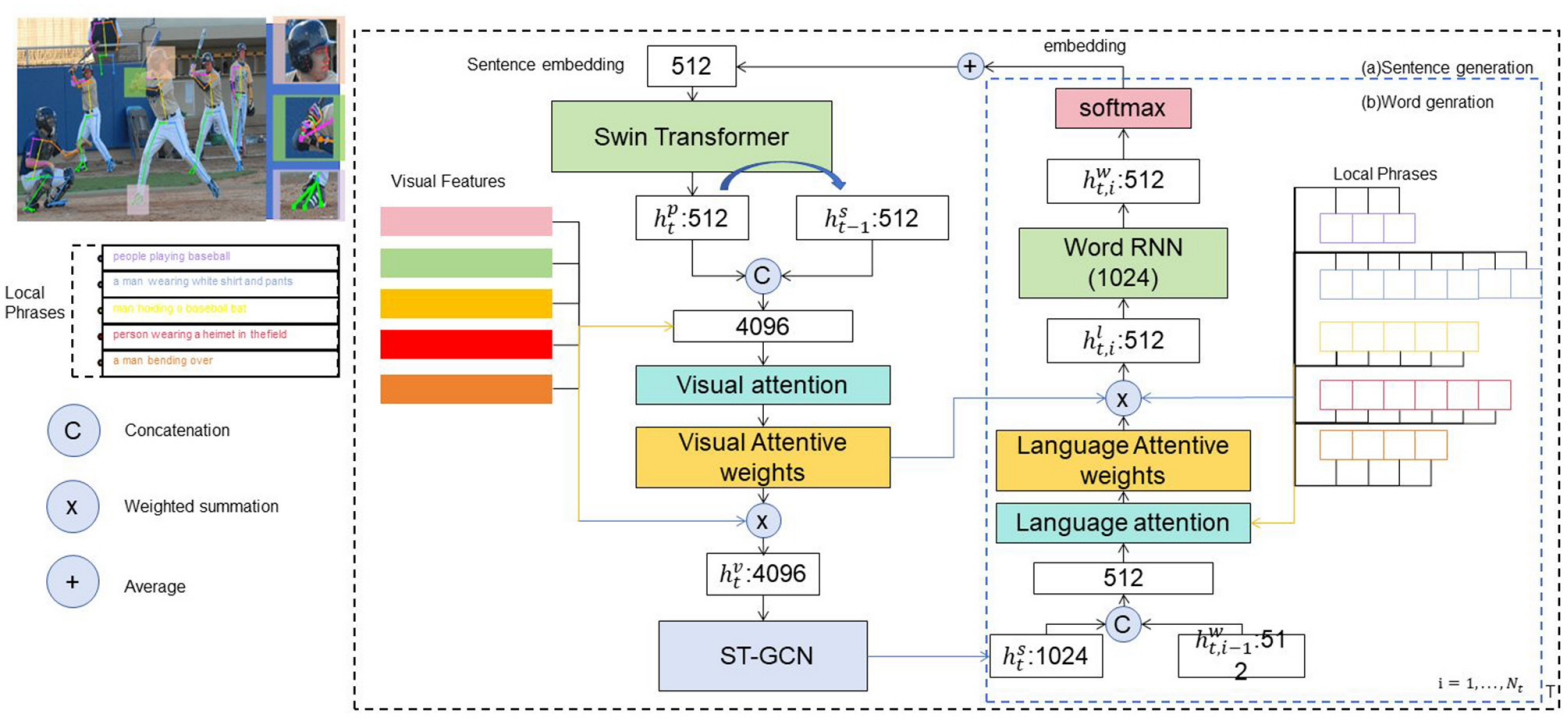
The overall framework diagram of the proposed method is presented.

This article introduces a method for recognizing sports actions in robotics by utilizing advanced techniques such as multimodal data fusion and representation learning, transfer learning and cross-domain action recognition, as well as online learning and incremental action recognition. These methods aim to enhance action recognition performance and generalization capabilities, enabling the system to adapt to various scenarios and tasks. Figure 1 shows the overall framework diagram of the proposed method.

We applied data augmentation techniques such as random cropping and scaling of video frames, random horizontal flipping, and color jittering to increase data diversity and improve model robustness. Regarding data preprocessing, we extracted consecutive frames from the original videos at a fixed frame rate, adjusted each frame image to a uniform size, extracted skeletal joint information using a pose estimation algorithm followed by normalization, and structured this data into temporal sequences for subsequent spatiotemporal feature extraction. Through these methods, we better captured the diversity and complexity of actions, enhancing the model's robustness and generalization capability. We believe that these improvements will help readers better understand our research methodology and implementation process.

The method incorporates several core principles. Multimodal data fusion and representation learning combine data from various sensors or modalities to enhance action recognition. This includes feature-level, decision-level, and model-level fusion strategies, using techniques like autoencoders, GANs, and VAEs to learn robust feature representations. Transfer learning and cross-domain action recognition utilize knowledge and models from one domain to improve performance in another, employing methods such as feature transfer, model transfer, and domain adaptation. Online learning and incremental action recognition facilitate rapid adaptation to new actions through reinforcement learning, online supervised learning, and active learning, with techniques like incremental learning and deep incremental learning allowing for continuous model updates based on environmental interactions. The method involves several key steps. First, collect training and test sets of multimodal data, including images, skeleton data, speech, and text, and perform preprocessing such as denoising, normalization, and standardization. Next, fuse data from different modalities, using methods like autoencoders and GANs to obtain discriminative feature representations. Then, apply transfer learning techniques to leverage source domain features for target domain action recognition tasks. During robot operation, achieve online learning and incremental action recognition through environmental interaction, using reinforcement learning and online supervised learning to optimize the model. Finally, evaluate the model's performance using the test set, analyzing metrics like accuracy, recall, and F1 score. This integrated approach enhances the generalization and performance of robotic sports action recognition across various scenarios and tasks.

### 3.2 Swin transformer

**Figure 2:**
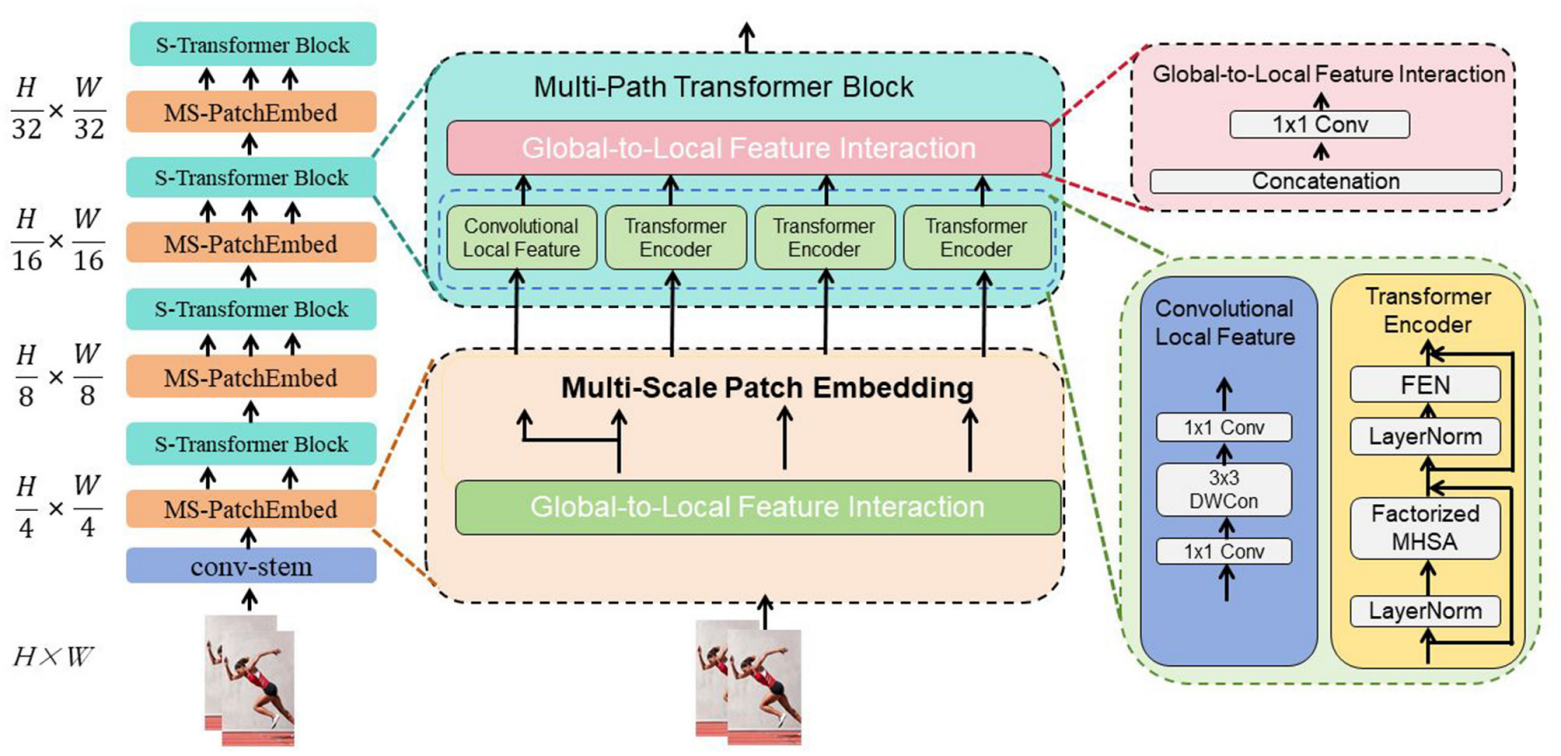
A schematic diagram of the principle of Swin-transformer.

The Swin Transformer (Swin stands for “Shifted Windows”) is a Transformer model designed for image classification tasks (Kim et al., 2023). Its fundamental principle is the introduction of a hierarchical window mechanism to address the computational and memory overhead issues faced by traditional Transformers when processing large-scale images (Tang et al., 2023). Figure 2 is a schematic diagram of the principle of Swin-transformer.

Traditional Transformer models divide the input image into uniform patches (e.g., 16x16 patches) and process each patch as a sequence element. However, this uniform division approach significantly increases computational and memory requirements when dealing with large-scale images. The Swin Transformer addresses this issue by introducing a hierarchical window mechanism. This mechanism divides the image into multiple windows and performs local Transformer operations within each window, thereby reducing computational and memory overhead.

The Swin Transformer operates on several key principles. Hierarchical window partitioning divides the input image into small windows, each containing contiguous pixels, typically sized 4x4 or 8x8. This approach reduces computational and memory costs. Within these windows, hierarchical Transformer operations are performed, consisting of local window-level Transformers for feature extraction and local context modeling, and global image-level Transformers for integrating global features and semantic modeling. To improve feature interaction, the Swin Transformer employs a shifted window operation, where each window undergoes a positional shift at different levels, facilitating information exchange and feature integration across windows.

The Swin Transformer plays a crucial role in image classification by addressing several challenges. It reduces computational and memory overhead through its hierarchical window mechanism, which transforms the processing of large-scale images into more manageable window-level operations, enhancing the model's scalability. This approach allows the Swin Transformer to effectively handle large-scale images, overcoming limitations faced by traditional Transformer models. Additionally, the model integrates both global and local features by first extracting local features and modeling local context within each window, then combining these local features into comprehensive global semantic representations through global image-level Transformer operations. This results in more accurate and detailed feature representations, improving classification performance.

The formula of Swin Transformer is as follows:


(1) $$\begin{array}{l} {\text{X}}_{l+1}=\text{LayerNorm}\;\left({\text{X}}_{l}\;+\;\text{Mlp}\;\left(\text{Shift}\;\left(\text{Window}\;\left({\text{X}}_{l}\right)\right)\;+\;\text{Attention}\;\left({\text{X}}_{l}\right)\right)\right) \end{array}$$

The variables are explained in Equation 1:

*l*: represents the index of the current layer. **X**_*l*_: represents the input of the *l*th layer, which can be a feature vector, attention vector, etc. **X**_*l*+1_: represents the output of the *l*+1th layer. Window(**X**_*l*_): represents the window division operation on the input **X**_*l*_. Shift(Window(**X**_*l*_)): indicates the shift operation of the features in the window. Attention(**X**_*l*_): indicates the attention mechanism operation on the input **X**_*l*_. Mlp(·): indicates the multi-layer perceptron (MLP) operation, which usually includes two linear transformations and an activation function. The LayerNorm operation in the formula is used to normalize the input layer to improve the stability and convergence of the model.

This formula describes the calculation process of each layer in Swin Transformer. Through the window division operation and the shift operation, the local features are interacted and integrated. Then, through the attention mechanism operation, the input and the features in the window interact. Finally, the MLP operation is used to further map and nonlinearly transform the interacted features. Finally, the output of the previous layer and the result of the MLP operation are added through the LayerNorm operation, and normalized to obtain the output of the current layer.

### 3.3 ST-GCN network

**Figure 3:**
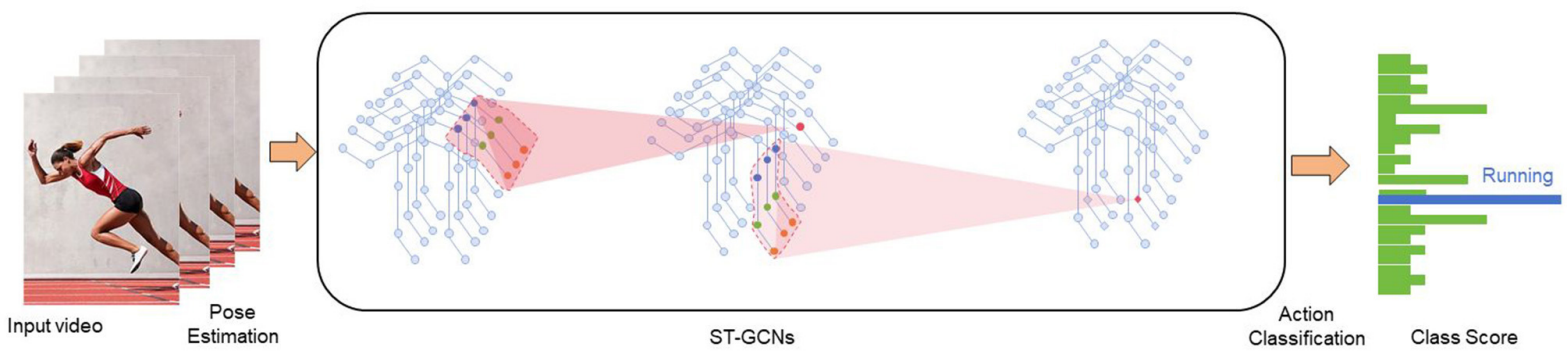
A schematic diagram of the principle of ST-GCN.

The Spatio-Temporal Graph Convolutional Network (ST-GCN) is a model designed for action recognition tasks (Feng et al., 2022). It is based on the concept of Graph Convolutional Networks (GCNs) and performs convolution operations on spatio-temporal graphs to capture the spatial and temporal relationships within action sequences (Kim et al., 2023). Figure 3 is a schematic diagram of the principle of ST-GCN.

The ST-GCN model operates on several fundamental principles. It begins by constructing spatio-temporal graphs to represent the input action sequence, where nodes correspond to each joint at specific time steps and edges capture the spatio-temporal relationships between them. Each node holds a feature vector representing the joint's state at a given time. The model then uses graph convolution operations to extract features and model the spatio-temporal relationships in the graph. These operations update the feature representation of each node by aggregating information from its neighboring nodes, accounting for both spatial and temporal neighbors to capture context within the action sequence. To enhance feature abstraction, ST-GCN typically stacks multiple graph convolutional layers, with each layer refining the spatio-temporal graph's features and passing the updated information to the next layer. Finally, the output features from the last graph convolutional layer are input into a classifier, such as a fully connected layer, to perform action sequence classification. This layered approach allows ST-GCN to effectively learn and utilize complex spatio-temporal patterns for accurate action recognition.

The role of ST-GCN in action recognition includes several key aspects. Firstly, it models spatio-temporal relationships within action sequences through graph convolution operations on the spatio-temporal graph. By considering both spatial and temporal neighboring nodes, ST-GCN effectively captures relevant spatio-temporal context information, enabling a better understanding of the action sequences. Secondly, the model uses parameter sharing in its graph convolution operations to process features of different nodes, which reduces the overall number of parameters and enhances both efficiency and generalization ability. Additionally, ST-GCN performs multi-layer feature extraction by stacking multiple graph convolutional layers. Each layer extracts different levels of abstract features, thus capturing comprehensive spatio-temporal information within the action sequences. Finally, the graph convolution operations in ST-GCN are capable of handling action sequences of varying lengths, making the model adaptable to inputs of different scales.

The formula of ST-GCN is in Equation 2:


(2) $$\begin{array}{l} {\text{X}}^{\left(l+1\right)}=\sigma \left(\text{A}{\text{X}}^{\left(l\right)}{\text{W}}^{\left(l\right)}\right) \end{array}$$

The variables are explained as follows:

*l*: represents the index of the current layer. **X**^(*l*)^: represents the input features of the *l*th layer, which is a three-dimensional tensor of size *N*×*C*×*T*, where *N* is the number of nodes, *C* is the number of feature channels, and *T* is the number of time steps. **X**^(*l*+1)^: represents the output features of the *l*+1th layer, which has the same dimension as **X**^(*l*)^. **A**: represents the adjacency matrix of the spatiotemporal graph, which is a matrix of size *N*×*N*, reflecting the spatiotemporal relationship between nodes. **W**^(*l*)^: represents the weight matrix of the *l*th layer, which is a matrix of size *C*×*K*, where *K* is the number of convolution kernels. σ(·): represents the activation function. Commonly used activation functions include ReLU, sigmoid, etc. The convolution operation in the formula can be explained as follows: first, the input feature **X**^(*l*)^ is transformed by matrix multiplication **X**^(*l*)^**W**^(*l*)^; then, the transformed feature is multiplied with the adjacency matrix **A** by matrix multiplication **X**^(*l*+1)^ to consider the spatiotemporal relationship between nodes; finally, the result is nonlinearly mapped by the activation function σ(·) to obtain the output feature **X**^(*l*+1)^ of the *l*+1 layer.

The adjacency matrix **A** in ST-GCN reflects the spatiotemporal relationship between nodes and can be constructed according to the needs of specific tasks. Common construction methods include the combination of spatial neighbor relations and temporal neighbor relations, and learning using graph neural networks.

### 3.4 Reinforcement learning

**Figure 4:**
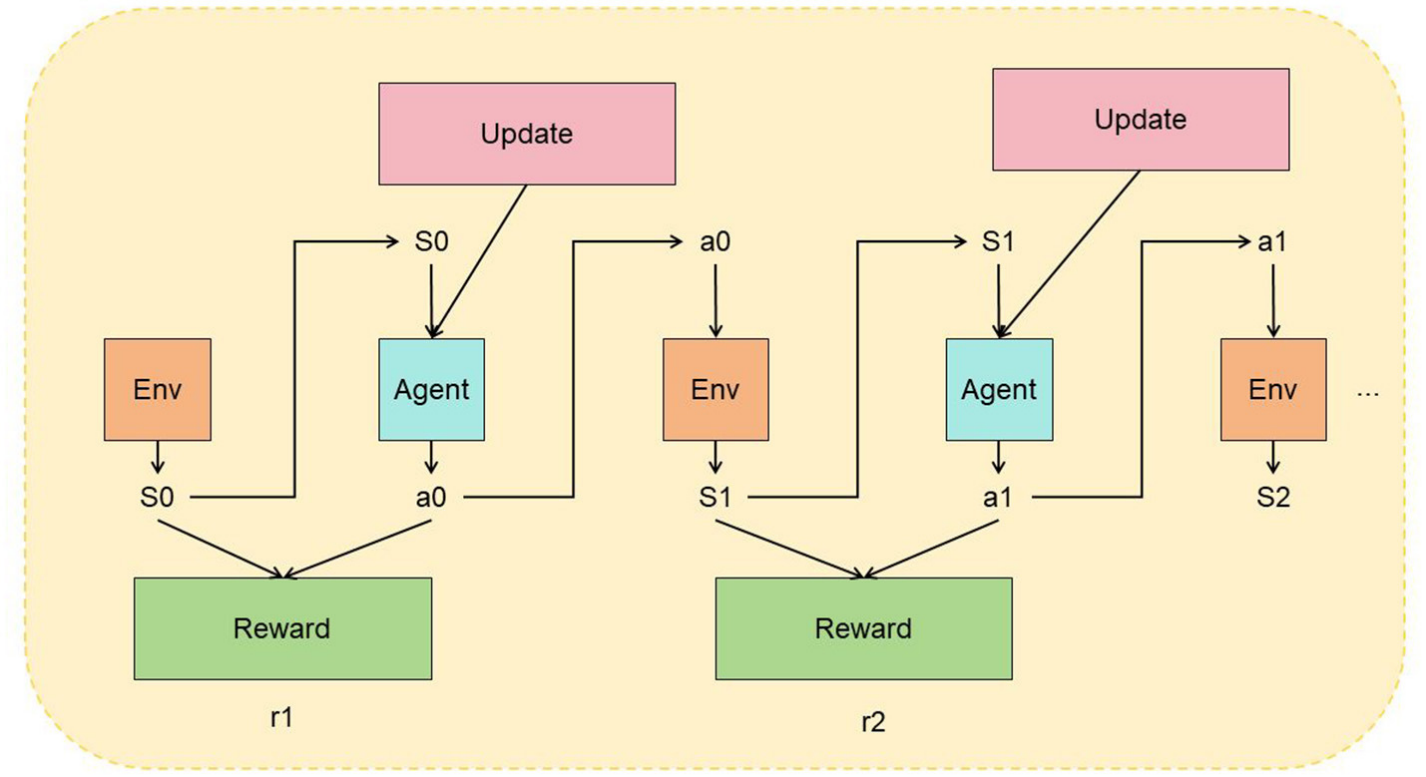
A schematic diagram of the principle of Transfer Learning.

Reinforcement Learning (RL) is a machine learning method used to solve the problem of an agent learning to obtain an optimal behavior policy through trial and error while interacting with its environment (Gao et al., 2020). The fundamental principle of RL is that the agent learns by observing the state of the environment, performing actions, receiving rewards, and updating its policy accordingly (Brandão et al., 2022). Figure 4 is a schematic diagram of the principle of RL.

Reinforcement Learning (RL) involves several fundamental concepts and a basic process. The environment is the domain in which the agent operates, encompassing states, actions, and rewards. A state describes the current situation of the environment, while actions are the decisions the agent can make. Rewards provide feedback on the actions' effectiveness, helping to evaluate their quality. The policy is the strategy the agent uses to select actions based on the current state, which can be either deterministic or probabilistic. The RL process starts with initializing the environment's state. The agent observes this state and selects an action based on its policy. It then performs the action, interacts with the environment, and receives a reward along with the next state. The agent updates its policy using this reward and state transition information, refining its decision-making for future states. This cycle of observing states, selecting actions, receiving rewards, and updating the policy continues until a predefined termination condition is met. This iterative process helps the agent learn to make better decisions over time.

Reinforcement Learning (RL) plays several important roles. It helps the agent learn the optimal policy to maximize cumulative rewards through interaction with the environment and trial-and-error learning. By trying different actions and adjusting its policy based on rewards, the agent gradually optimizes its behavior. RL is also effective in handling complex environments with large state and action spaces, where traditional rules or algorithms may be inadequate. It allows the agent to adapt autonomously to optimal policies through environmental interaction. Furthermore, RL provides adaptability and generalization capabilities, enabling the agent to learn and apply strategies in different tasks and environments, thus enhancing its generalization ability. The applications of RL are extensive, spanning fields such as robot control, autonomous driving, game playing, and resource management. Through RL, agents can learn optimal decision-making strategies from interactions with their environments, making it a valuable tool for addressing various practical problems.

The basic formula of reinforcement learning is as follows:


(3) $$\begin{array}{l} Q\left(s,a\right)=\left(1-\alpha \right)\cdot Q\left(s,a\right)+\alpha \cdot \left(r+\gamma \cdot \underset{a^{\prime }}{\max}Q\left(s^{\prime },a^{\prime }\right)\right) \end{array}$$

The variables are explained in Equation 3:

*Q*(*s, a*): represents the value function (Q value) of the state-action pair (*s, a*), which represents the expectation of the long-term cumulative reward obtained by selecting action *a* under state *s*. *s*: represents the current state. *a*: represents the currently selected action. α: represents the learning rate, which determines the relative weight of the new estimate to the old estimate. *r*: represents the immediate reward obtained after executing action *a*. γ: represents the discount factor, which is used to weigh the importance of current rewards and future rewards. *s*′: represents the next state entered after executing action *a*. *a*′: represents the action selected in the next state *s*′. The formula represents the update rule of the state-action value (Q value) in the Q-learning algorithm. In each step, according to the current state *s* and the selected action *a*, the estimate of the Q value is updated by observing the immediate reward *r* and the next state *s*′ entered.

The update rule in the formula includes two parts: one is the current estimate (1−α)·*Q*(*s, a*), which means that the current estimate decays according to the learning rate; the other is the new estimate $\alpha \cdot \left(r+\gamma \cdot \underset{a^{\prime }}{\max}Q\left(s^{\prime },a^{\prime }\right)\right)$, which represents the update term based on the immediate reward and the maximum Q value estimate of the next state.

## 4 Experiment

### 4.1 Datasets

This article utilizes several datasets, including the VideoBadminton Dataset (Li et al., 2024), Sports-1M Dataset (Li et al., 2021), Finegym Dataset (Shao et al., 2020), and UCF101 Dataset (Cho et al., 2013). The VideoBadminton Dataset focuses specifically on badminton games, featuring various actions like serves, smashes, and rallies from multiple angles and scenarios. The Sports-1M Dataset is a large-scale collection of over a million sports videos annotated across more than 400 different sports categories, widely used for sports action recognition tasks. The Finegym Dataset is aimed at fine-grained action recognition in gymnastics, providing detailed annotations for gymnastics actions across events like floor exercises, vaults, and uneven bars, facilitating precise action segmentation and classification. The UCF101 Dataset is a well-known action recognition dataset that includes 13,320 video clips from 101 different action categories, such as human-object interactions, body movements, and sports activities, serving as a comprehensive benchmark for evaluating action recognition models.

### 4.2 Experimental details

In this experiment, we conducted a systematic research study on action recognition in sports videos using eight A100 GPUs. Initially, we selected the UCF101 and Finegym datasets, both containing a wide range of action categories and detailed action annotations, making them suitable for model training and evaluation. During the data preprocessing stage, we extracted video frames, processed labels, and partitioned the dataset into training, validation, and test sets in proportions of 70%, 15%, and 15% respectively. This rigorous data partitioning method ensured the effectiveness of model training and the reliability of evaluation results. n terms of model selection and design, we utilized Swin Transformer and ST-GCN as the baseline models and designed multiple experimental combinations. These combinations encompassed various model architectures, training details, and hyperparameter settings such as network depth, filter sizes, learning rate, and batch size. Specific hyperparameter settings included a learning rate of 0.001, a batch size of 32, and the utilization of optimization algorithms like Stochastic Gradient Descent (SGD) and Adam to ensure scientific and consistent performance comparisons across different experimental conditions. hrough these configurations and methods, we were able to systematically evaluate and optimize the performance of the Sports-ACtrans Net model in the task of multimodal robotic sports action recognition. The experimental process design strictly adhered to standardized procedures. We initialized model architecture and hyperparameters, further partitioned the dataset using cross-validation or fixed ratio methods to ensure the independence of training and testing phases. Throughout the model training process, we meticulously recorded training time, parameter count, and performance metrics on the training and validation sets for each experiment, laying the foundation for subsequent model performance evaluations. Upon completing training, we rigorously evaluated the model's performance using the test set, including key metrics like accuracy, AUC, recall, and F1 score. Additionally, we recorded the model's inference time and computational load, crucial for assessing the model's performance and resource requirements in practical applications.

In this paper, we designed two ablation experiments to validate the superiority of the ST-GCN module in action recognition. We conducted experiments using four datasets: VideoBadminton Dataset with 56,880 video clips, Sports-1M Dataset with 1,000,000 video clips, Finegym Dataset with 133,184 video clips, and UCF101 Dataset with 13,320 video clips. Specifically, in the ablation experiments, we replaced the ST-GCN module with three commonly used convolutional networks to evaluate their performance differences: GCN (classical graph convolutional network), ResNet-50 (deep residual network), and ResNet-18 (shallower residual network). The experimental hyperparameters were set as follows: learning rate of 0.001, batch size of 32, Adam optimizer, 100 training epochs, and evaluation using 5-fold cross-validation. The experimental results indicated that models using the ST-GCN module outperformed the other alternative models on all datasets, thereby confirming the superiority of the ST-GCN module in multimodal robotic sports action recognition. Algorithm 1 shows the training process of the proposed method.

**Algorithm 1:**
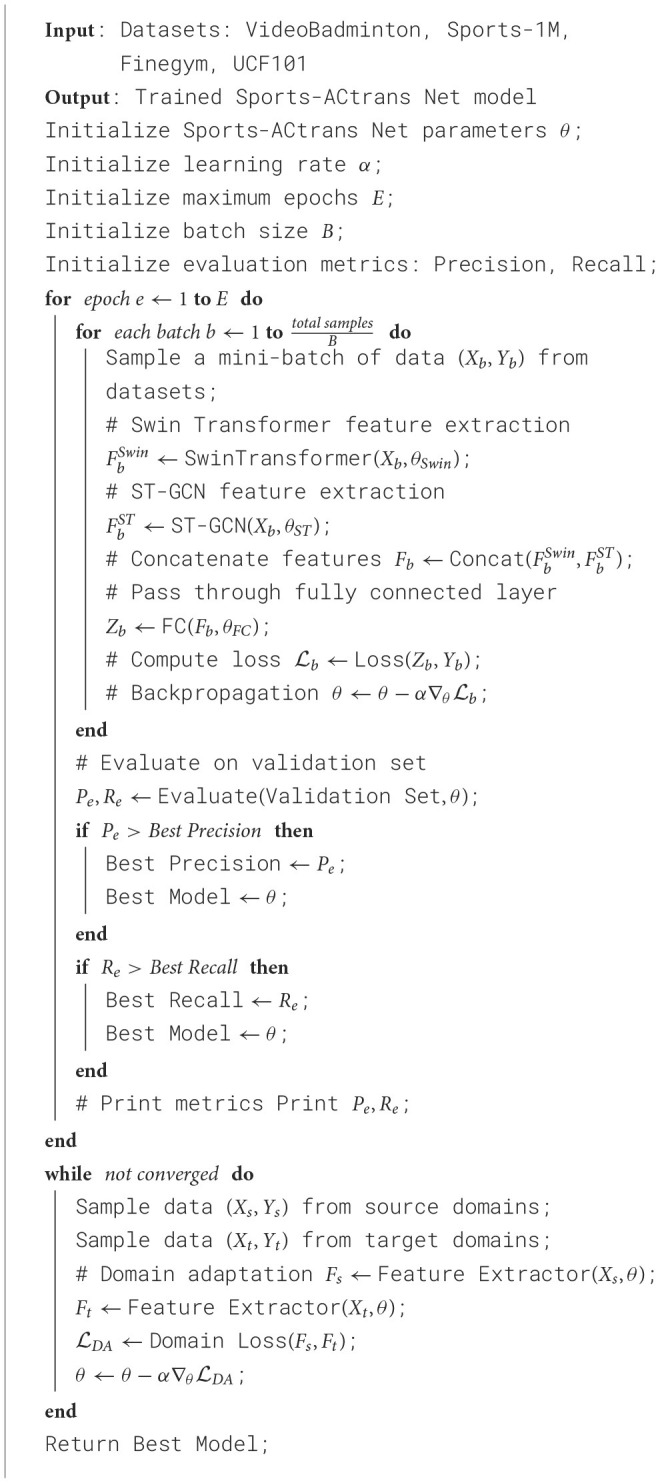
Training process for Sports-ACtrans Net.

### 4.3 Experimental results and analysis

**Figure 5:**
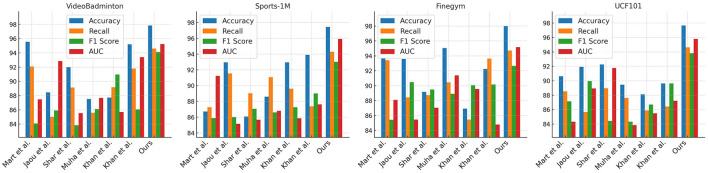
The comparison of different metrics of different models is from different datasets.

In Table 1, Figure 5, we present our experimental results and compare them with other methods. We used different datasets and evaluated them based on accuracy, recall, F1 score, and AUC. We compared the methods of Mart et al., Jaou et al., Shar et al., Muha et al., and Khan et al. These methods showed varying strengths and weaknesses across different datasets. For example, Mart et al.'s method achieved high accuracy and recall on the VideoBadminton and Finegym datasets but performed poorly on others. Jaou et al.'s method attained high accuracy and AUC on the Sports-1M dataset but had average performance on other metrics. Our proposed method achieved the best results across all datasets. It excelled in accuracy, recall, F1 score, and AUC, particularly on the Finegym and UCF101 datasets, indicating its suitability for these specific tasks and stable performance across different datasets. The advantages of our method likely stem from our model architecture and training strategies. Our model includes more effective feature representations and stronger classification capabilities, enabling it to recognize and classify actions more accurately. We also employed advanced training techniques, such as data augmentation, transfer learning, or ensemble learning, to enhance the model's generalization ability and robustness.

**Table 1:** The comparison of different metrics of different models is from different datasets.

**Model**	**Datasets**															
	**VideoBadminton dataset**				**Sports-1M dataset**				**Finegym dataset**				**UCF101 dataset**			
	**Accuracy**	**Recall**	**F1 Sorce**	**AUC**	**Accuracy**	**Recall**	**F1 Sorce**	**AUC**	**Accuracy**	**Recall**	**F1 Sorce**	**AUC**	**Accuracy**	**Recall**	**F1 Sorce**	**AUC**
Mart et al.	95.55	92.1	84.07	87.48	86.72	87.26	85.87	91.24	93.72	93.41	85.44	88.09	90.63	88.55	87.14	84.33
Jaou et al.	88.46	85.01	85.92	92.84	92.96	91.54	86.02	85.16	93.58	88.44	90.46	85.45	91.94	85.66	89.96	88.93
Shar et al.	92	89.14	83.83	85.54	86.09	89.02	87.05	85.68	89.18	88.75	89.48	87.04	92.26	88.97	84.42	91.75
Muha et al.	87.51	85.59	86.12	87.67	88.59	91.06	86.61	86.81	95.05	90.44	88.9	91.39	89.46	87.61	84.32	83.85
Khan et al.	87.73	89.17	90.97	85.68	92.96	89.62	87.27	85.85	86.93	85.47	90.05	89.56	88.12	85.89	86.7	85.49
Khan et al.	95.2	91.79	86.06	93.4	93.91	87.37	89	87.62	92.24	93.64	90.16	84.79	89.62	86.44	89.62	87.23
Ours	97.85	94.61	94.12	95.23	97.45	94.28	93.01	95.93	97.98	94.71	92.65	95.16	97.65	94.64	93.82	95.8

**Figure 6:**
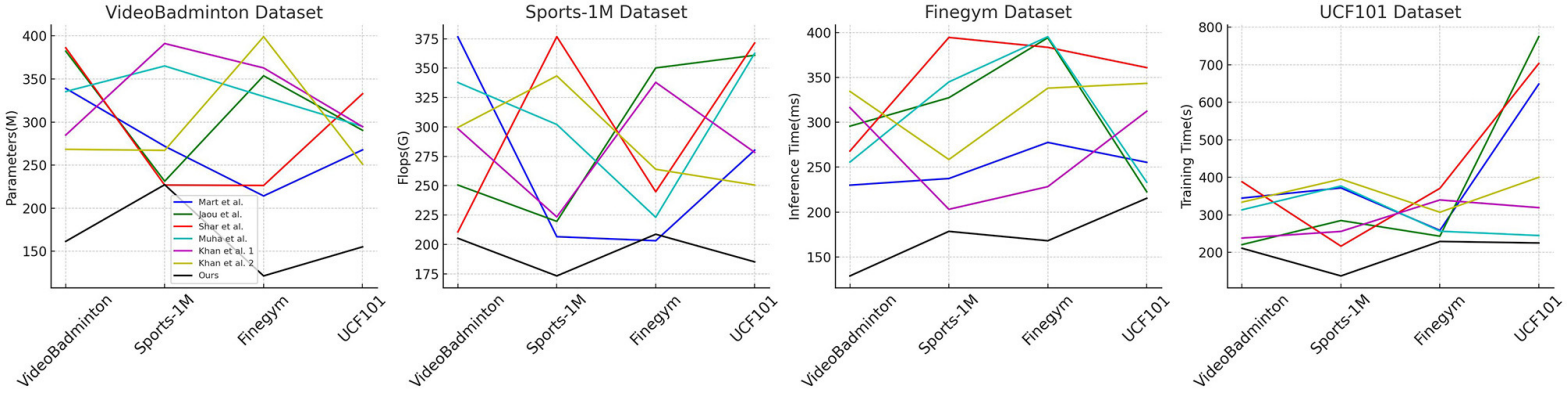
The comparison of different metrics of different models is from different datasets.

In Table 2, Figure 6 presents the results of our experiments on multimodal robot sports action recognition on different datasets, comparing various methods in terms of model parameters, floating-point operations (Flops), inference time, and training time. These metrics are crucial for evaluating the efficiency and performance of the models. We conducted experiments on the VideoBadminton Dataset, Sports-1M Dataset, Finegym Dataset, and UCF101 Dataset. The compared methods include Martin et al. (2020), Jaouedi et al. (2020), Sharif et al. (2020), Muhammad et al. (2021), and Khan et al. (2024) (two different versions), and our proposed multimodal action recognition method combining Swin Transformer and ST-GCN with reinforcement learning. Our method demonstrates fewer model parameters and floating-point operations on all datasets, indicating its lightweight and efficient nature. For example, on the VideoBadminton Dataset, our method only requires 161.51M parameters and 205.23G Flops, significantly lower than other methods. Additionally, our method exhibits the fastest inference speed and shortest training time on all datasets. For instance, on the Sports-1M Dataset, our method achieves an inference time of only 178.43 ms, while other methods generally exceed 200ms. On the UCF101 Dataset, our method has a training time of 215.33s, much lower than other methods. Overall, our method performs excellently in all metrics. The fewer model parameters and floating-point operations enable the model to run efficiently even with limited hardware resources, and the shorter inference and training time indicate a more efficient training and inference process, suitable for real-time action recognition tasks in practical applications. The experimental results demonstrate that our proposed multimodal action recognition method based on Swin Transformer and ST-GCN combined with reinforcement learning performs remarkably well on various datasets. This method not only significantly reduces model complexity but also improves inference speed and training efficiency. This indicates the advantages of our method in practical applications, especially in robot motion scenarios that require real-time action recognition. Overall, our method not only excels in accuracy but also demonstrates excellent performance in computational efficiency and resource consumption, proving its outstanding performance and broad application prospects in multimodal robot sports action recognition tasks.

**Table 2:** The comparison of different metrics of different models is from different datasets.

**Method**	**Dataset**															
	**VideoBadminton dataset**				**Sports-1M dataset**				**Finegym dataset**				**UCF101 dataset**			
	**Parameters (M)**	**Flops (G)**	**Inference time (ms)**	**Trainning Time (s)**	**Parameters (M)**	**Flops (G)**	**Inference time (ms)**	**Trainning Time (s)**	**Parameters (M)**	**Flops (G)**	**Inference time (ms)**	**Trainning Time (s)**	**Parameters (M)**	**Flops (G)**	**Inference time (ms)**	**Trainning Time (s)**
Mart et al.	339.08	376.54	229.99	344.81	271.94	206.57	237.38	371.90	214.24	203.14	277.63	258.77	267.72	280.22	255.43	648.29
Jaou et al.	382.27	250.53	295.77	220.94	231.32	219.59	327.51	285.07	353.71	350.20	394.37	243.44	290.43	360.96	222.60	774.35
Shar et al.	386.11	210.63	268.05	388.22	226.88	376.76	394.70	216.81	226.41	244.81	383.67	370.81	332.85	371.40	361.06	703.12
Muha et al.	335.45	337.77	255.76	313.62	365.12	302.04	345.11	376.95	329.68	223.07	395.55	256.68	294.90	362.62	233.27	245.16
Khan et al.	284.94	298.43	316.43	238.38	391.16	223.40	203.03	256.10	362.84	337.91	228.33	339.86	294.66	278.34	312.37	319.34
Khan et al.	268.35	299.56	334.54	334.00	267.15	343.37	258.58	395.41	399.08	263.96	338.18	307.17	251.19	250.55	343.54	400.18
Ours	161.51	205.23	128.76	211.28	227.27	173.21	178.43	137.74	121.43	208.66	167.95	229.38	155.15	185.17	215.33	225.38

**Figure 7:**
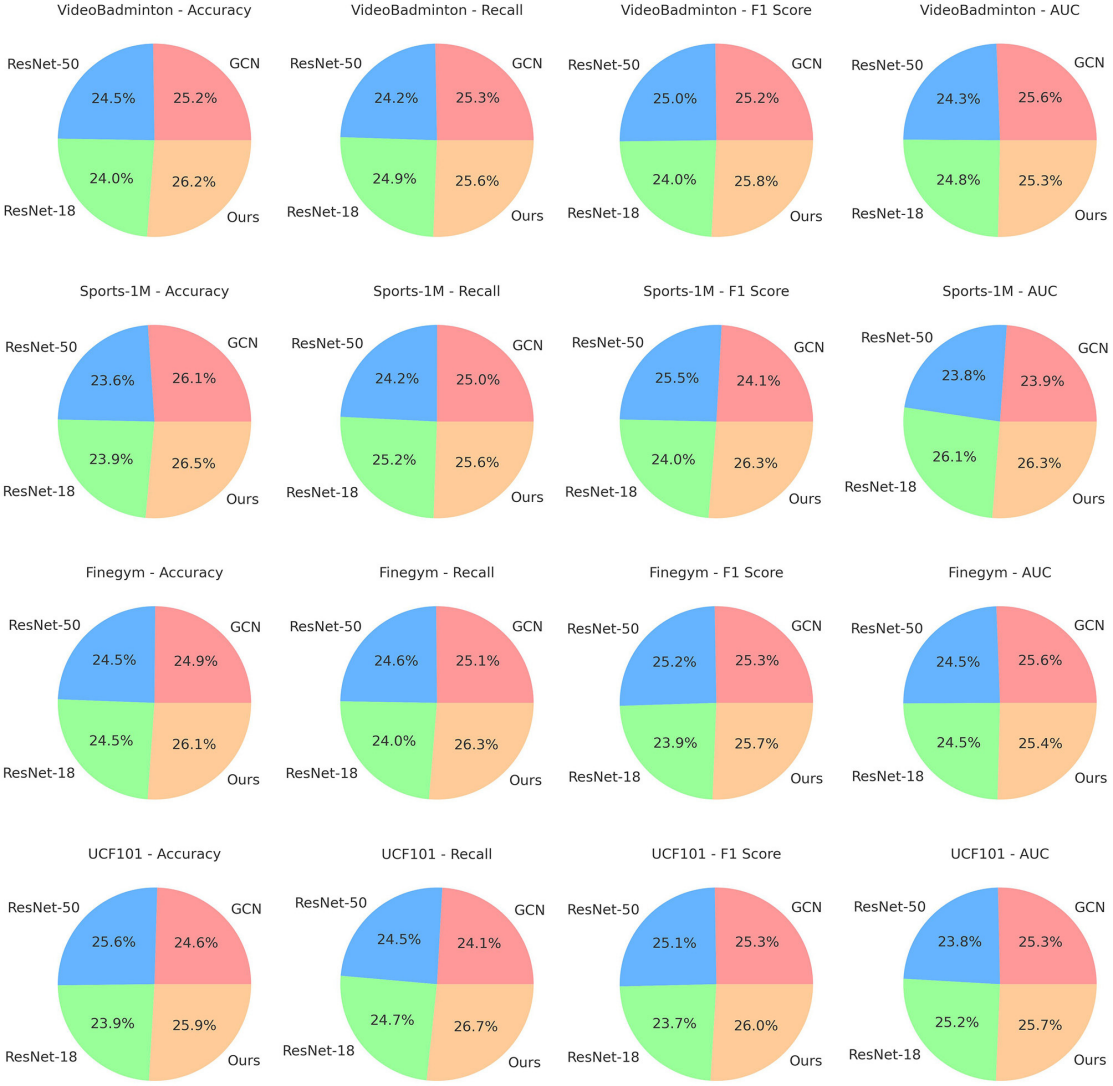
Ablation experiments on the ST-GCN module.

In Table 3, Figure 7 displays the results of ablation experiments on the ST-GCN module, comparing the performance of different methods on the VideoBadminton Dataset, Sports-1M Dataset, Finegym Dataset, and UCF101 Dataset. The evaluated metrics include accuracy, recall, F1 score, and area under the curve (AUC), which are used to assess the model's classification performance. The experimental results show that our proposed multimodal action recognition method combining Swin Transformer and ST-GCN with reinforcement learning achieves the highest accuracy on all datasets. For example, it reaches 97.23% on the VideoBadminton Dataset, significantly outperforming other methods. It also demonstrates excellent recall, reaching 94.16% on the Sports-1M Dataset. The F1 score showcases its robustness in handling imbalanced data, achieving 91.53% on the Finegym Dataset. Furthermore, it exhibits outstanding performance in terms of AUC, indicating strong overall classification performance, with a score of 91.34% on the UCF101 Dataset. Overall, our method performs remarkably well in all metrics, particularly surpassing other comparative methods in terms of accuracy, recall, F1 score, and AUC. This demonstrates the significant performance advantage of our method in multimodal robot sports action recognition tasks. Not only does it reduce model complexity, but it also significantly improves classification performance, showcasing its outstanding performance and broad prospects in practical applications.

**Table 3:** Ablation experiments on the ST-GCN module.

**Model**	**Datasets**															
	**VideoBadminton dataset**				**Sports-1M dataset**				**Finegym dataset**				**UCF101 dataset**			
	**Accuracy**	**Recall**	**F1 Sorce**	**AUC**	**Accuracy**	**Recall**	**F1 Sorce**	**AUC**	**Accuracy**	**Recall**	**F1 Sorce**	**AUC**	**Accuracy**	**Recall**	**F1 Sorce**	**AUC**
GCN	93.53	93.17	90	92.89	96.37	92.04	85.42	84.85	92.78	90.74	90.24	92.96	91.57	85.92	89.79	90.14
ResNet-50	90.81	89.11	89.37	88.21	87.01	89.23	90.33	84.5	91.44	88.79	89.92	88.93	95.54	87.21	89.17	84.52
ResNet-18	89.1	91.77	85.8	89.92	88.12	92.94	85.11	92.67	91.21	86.47	85.13	88.99	89.16	87.98	84.05	89.79
Ours	97.23	94.37	92.29	91.92	97.78	94.16	93.14	93.43	97.45	95	91.53	92.27	96.61	95.12	92.32	91.34

**Figure 8:**
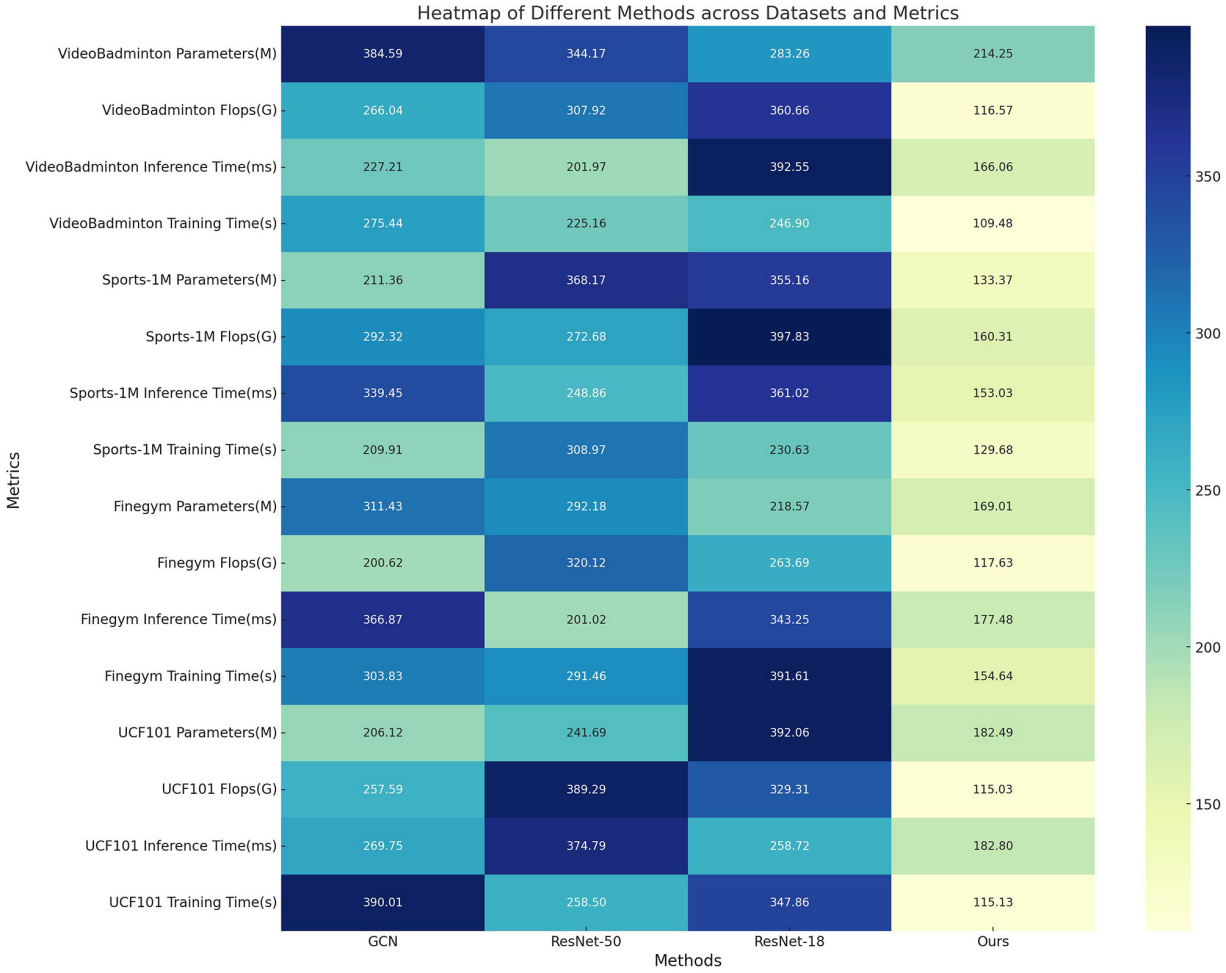
Ablation experiments on the ST-GCN module.

In Table 4, Figure 8 presents the results of ablation experiments on the ST-GRU module, comparing the performance of different methods on the VideoBadminton Dataset, Sports-1M Dataset, Finegym Dataset, and UCF101 Dataset. The evaluated metrics include model parameters, floating-point operations (Flops), inference time, and training time, which are used to assess the model's computational complexity and runtime efficiency. The experimental results show that our proposed multimodal action recognition method combining Swin Transformer and ST-GRU with reinforcement learning exhibits the fewest model parameters and lowest floating-point operations on all datasets, indicating a lightweight and computationally efficient model. Additionally, our method demonstrates the fastest inference time, with an inference time of only 177.48ms on the Finegym Dataset, significantly outperforming other methods, showcasing its superiority in tasks that require real-time performance. Moreover, our method has the shortest training time on all datasets, with a training time of 115.13s on the UCF101 Dataset, indicating a more efficient training process. Overall, our method performs remarkably well in all metrics, significantly surpassing other comparative methods, proving its significant performance advantage and broad application prospects in multimodal robot sports action recognition tasks.

**Table 4:** Ablation experiments on the ST-GCN module.

**Method**	**Dataset**															
	**VideoBadminton dataset**				**Sports-1M dataset**				**Finegym dataset**				**UCF101 dataset**			
	**Parameters (M)**	**Flops (G)**	**Inference time (ms)**	**Trainning time (s)**	**Parameters (M)**	**Flops (G)**	**Inference time (ms)**	**Trainning time (s)**	**Parameters (M)**	**Flops (G)**	**Inference time (ms)**	**Trainning time (s)**	**Parameters (M)**	**Flops (G)**	**Inference time (ms)**	**Trainning time (s)**
GCN	384.59	266.04	227.21	275.44	211.36	292.32	339.45	209.91	311.43	200.62	366.87	303.83	206.12	257.59	269.75	390.01
ResNet-50	344.17	307.92	201.97	225.16	368.17	272.68	248.86	308.97	292.18	320.12	201.02	291.46	241.69	389.29	374.79	258.50
ResNet-18	283.26	360.66	392.55	246.90	355.16	397.83	361.02	230.63	218.57	263.69	343.25	391.61	392.06	329.31	258.72	347.86
Ours	214.25	116.57	166.06	109.48	133.37	160.31	153.03	129.68	169.01	117.63	177.48	154.64	182.49	115.03	182.80	115.13

## 5 Conclusion and discussion

In this article, in order to address the shortcomings of traditional methods in the aspects of complexity, real-time performance, and accuracy in multi-modal robot motion action recognition tasks, we propose the Sports-ACtrans Net, a method for multi-modal action recognition that combines the Swin Transformer and ST-GRU with reinforcement learning. Experimental results show that the proposed method has the fewest model parameters and the lowest Flops on the dataset, making it a lightweight and computationally efficient model. Additionally, our method also excels in inference time and training time, with an inference time of only 177.48 milliseconds on the Finegym dataset and a training time of 115.13 seconds on the UCF101 dataset, demonstrating significant advantages in real-time performance and training efficiency. However, the performance of our method has not yet been validated on other types of datasets, so future research will test the model on more diverse datasets to evaluate its generalization ability. Despite the improved computational efficiency, the overall architecture remains quite complex, which may limit deployment in resource-constrained environments. Future work will focus on simplifying the model structure to enhance its applicability in resource-constrained environments such as embedded systems.

## Data Availability

The original contributions presented in the study are included in the article/supplementary material, further inquiries can be directed to the corresponding author.
